# Comparison of the different isoforms of vitamin e against amyloid beta-induced neurodegeneration

**DOI:** 10.55730/1300-0152.2625

**Published:** 2022-08-16

**Authors:** Alp Yiğit ÖZDEMİR, Esin AKBAY, Mehmet Ali ONUR

**Affiliations:** Hacettepe University, Department of Biology, Çankaya, Ankara, Turkey

**Keywords:** Amyloid beta, Neurodegeneration, A-tocopherol, A-tocotrienol

## Abstract

Neurodegeneration is the progressive loss of structure or function of neurons. Amyloid beta oligomers and aggregates have been linked to neurodegeneration. While previous studies have suggested that dietary α-tocopherol intake can prevent amyloid beta aggregation and protect the brain against neurotoxicity, other research, however, indicated that tocotrienol forms might be used as an alternate agent against this kind of degeneration. In the presented research, we compared the in vitro protective effects of α-tocopherol and α-tocotrienol. In this context, we formed an in vitro neurodegeneration model with primary isolated neurons and measured α-tocopherol’s and α-tocotrienol’s protective effects. As a result, α-tocopherol and α-tocotrienol prevent the degeneration of neurons. Moreover, α-tocopherol and α-tocotrienol regulated the neuron’s calcium channels mechanism by decreasing the expression of the calcium channel alpha 1C subunit. We also observed that the amount of amyloid beta accumulation in the extracellular matrix decreased with the application of these isoforms. In specific time points, α-tocopherol and α-tocotrienol differ in terms of protective effects. In conclusion, it could be interpreted that, in more extended periods, α-tocotrienol could be a significant protective agent against amyloid beta-induced neurodegeneration, and it can be used as an alternative to other protective agents, especially α-tocopherol.

## 1. Introduction

Neurodegeneration means the deterioration of neurons for several reasons. It may occur due to various causes, including the accumulation of protein aggregates such as amyloid beta (Aβ) or tau, oxidative stress, and genetic mutations (Hoeijmakers, 2009; Chang et al., 2018). Pathologies of neurodegenerative diseases like Alzheimer’s are generally characterized by the accumulation of insoluble extracellular aggregates such as Aβ aggregates in specific brain regions (Goedert, 2007). Aβ is a protein fragment caused by the wrong process of a protein called amyloid precursor protein (APP). Different Aβ forms take names such as Aβ40 or Aβ42 based on the number of amino acids they contain. Forms such as Aβ42 are much more toxic than other forms of Aβ for the brain (Chen et al., 2017; Rudajev and Novotny, 2020). Previous studies show that Aβ disturbs cell membrane functions, increases protein oxidation, creates oxidative stress, and harms the mechanisms that regulate calcium channel influx in the neuronal cells (Huang et al., 1999, Sciacca et al., 2018). Aβ oligomers cause mitochondrial dysfunction in neurons and induce oxidative stress, resulting in a large calcium influx and increased neuronal toxicity (Canevari et al., 2004). Studies show that lipophilic antioxidants such as vitamin E could be used against neurogenerative disorders like Alzheimer’s (Chang et al., 2018).

Vitamin E, an essential element for nutrition, is produced only by photosynthetic organisms. It affects many specific biological functions such as gene expression, certain signaling pathways, and cell proliferation. It also shows protective and curative effects against oxidative stress and acts like an antioxidant in the body (Grimm et al., 2005; Sen et al., 2006; Gugliandolo et al., 2019). There are eight isoforms of vitamin E, four tocopherols, and four tocotrienols. A-tocotrienol (A-T3) and α-tocopherol (A-T) are abundant in many herbal products consumed, especially vegetable oils (Sen et al., 2006; Ulatowski and Manor, 2015). Vitamin E shows its neuroprotective effects by preventing cell death resulting from Aβ-induced protein oxidation and accumulation of reactive oxygen species (ROS) (Yatin et al., 2000). Another study also showed that vitamin E could induce the nonamyloidogenic pathway of APP and modulate the autophagic flux in the brain (Gugliandolo et al., 2019). Apart from protecting cells against lipid peroxidation, vitamin E plays a critical role in maintaining neurological health, and its deficiency may cause conditions such as cellular atrophy and decreased branching of Purkinje neurons (Grimm et al., 2005; Ulatowski et al., 2013; Ulatowski and Manor, 2015; Gugliandolo et al., 2019). Some of the previous studies suggested that although A-T and A-T3 show similar effects at some levels against neurodegeneration, they may have different effects in areas such as preventing Aβ accumulation (Grimm et al., 2016; Ibrahim et al., 2021). As stated in another study, A-T also provides a therapeutic effect against oxidative damage in hippocampal cells by being involved in calcium metabolism (Crouzin et al., 2007). However, the effects of A-T and A-T3 on the mechanisms regulating calcium channel flux induced by Aβ have not been studied before, to our knowledge. Besides, the effects of protective agents such as A-T and A-T3 on the metabolism of Aβ-induced neurodegeneration are not fully understood and continue to be studied (Chang et al., 2018; Gugliandolo et al., 2019; Ibrahim et al., 2021; Yatin et al., 2000). Considering this, we examined the protective effects of A-T and A-T3 against Aβ-induced damage within the scope of the study. To acquire reliable results, we used cells obtained from primary neuron culture, which show a high degree of similarity to human neurons, and formed a neurodegeneration model. Then, we examined A-T and A-T3 against Aβ-induced neurodegeneration and compared them with each other in terms of cytotoxicity, apoptosis, necrosis, Aβ aggregation value, and expression of Beta-secretase 1 (BACE1) and Voltage-dependent calcium channel alpha 1C subunit (Cacna1c) genes.

## 2. Materials and methods

### 2.1. Materials and chemicals

Amyloid β protein fragment 1–42 (CAS number: 107761-42-2), α-tocopherol (CAS number: 10191-41-0), D-α-tocotrienol (CAS number: 1721-51-3), cytosine β-Darabinofuranoside (CAS number: 147-94-4), L-glutamic acid (CAS: number: 56-86-0), L-glutamine (CAS number: 56-85-9), penicillin-streptomycin, (P/S) (CAS number: 3810-74-0), Congo red (CAS Number: 573-58-0), 3-(4,5-dimethylthiazol-2-yl)-2,5-diphenyltetrazolium bromide (MTT) (CAS Number: 298-93-1), methyl blue (CAS number: 28983-56-4), neutral red solution (0.33%) (CAS number: 553-24-2), TRI Reagent^®^ (MDL number: MFCD00213058), Triton™ X-100 (CAS number: 9002-93-1), acridine orange (AO) solution (CAS Number: 65-61-2), and propidium iodide (PI) solution (CAS number: 25535-16-4) were obtained from Sigma-Aldrich (Germany). DMEM - F12 W/ stable glutamine W/ 15 MM Hepes (DMEM-F12) (Cat number: L0092-500), trypsin-EDTA (0.25%), phenol red (Cat number: 25200056), and Fetal Bovine Serum (FBS) (Cat number S1810-500) were purchased from Biowest MO, USA. Neurobasal™ Medium (Cat number: 21103049), B-27™ Plus supplement (50X) (Cat number: A3582801), 2-mercaptoethanol (50 mM) (Cat number: 31350010), and poly-D-lysine (PDL) (Cat number: A3890401) were purchased from Thermo Fisher Scientific, MA, USA. DAPI staining solution (ab228549), goat anti-mouse IgG H&L (Texas Red ^®^) (ab6787), and anti-MAP2 antibody [AP-20] (ab11268) were purchased from Abcam, Cambridge, UK. All chemicals and solvents were obtained as cell culture grade. *Rattus norvegicus* type Wistar albino rats were purchased from the Laboratory Animals Raising and Experimental Research Center (GÜDAM), Ankara, Turkey.

### 2.2. Preparation of experimental animals and obtaining embryos by surgical method

Embryos for use in primary neuron culture were obtained by mating three female rats with a male rat. In this study, we used a total of seven (six female and one male) rats within the framework of the ethical permission provided by the Hacettepe University Animal Experiments Local Ethics Committee (decision number: 52338575-68). After mating, vaginal plaque observation was performed to determine the exact time of pregnancy, and rats with vaginal plaque formation were considered starting day of pregnancy. We applied daily care and controls to the rats during their pregnancy. Pregnant rats were euthanized by cervical dislocation on the 17th or 18th days. Subsequently, the sacrificed rats were treated with 70% alcohol solution to ensure disinfection.

The embryos extracted from pregnant rats by the surgical method were transferred to 100 mm diameter glass petri dishes filled with 10 mL of the ice-cold transfer solution containing 1.5% (g/mL) sucrose and 0.6% (g/ mL) d-glucose. In order to keep cells alive by slowing their metabolism, we performed all surgical procedures on the embryos on ice. The brains were isolated under a dissecting microscope (Zoom 2000, Leica Camera, USA). The cortex regions of the isolated brains were then separated and cleared from meninges. Mechanical dissection of the cortices was carried out in a petri dish full of ice-cold transfer solution using a sterile scalpel. Dissected cortex pieces were then transferred to 15 mL tubes with 10 mL of transfer solution to apply primary neuron isolation methods.

### 2.3. Primary cortical neuron isolation and culture

For isolating neurons, we have followed the primary neuronal isolation methods described previously (Yatin et al., 2000). In this context, cortex pieces were washed by centrifugation twice at 1000 rpm for 1 min. Then, the pellet was separated and treated with 0.5 mL of 1X trypsin and 4.5 mL of transfer solution in a 37 °C water bath to break down the protein bonds in the tissues. At the end of the incubation with trypsin, the pellet was washed two times with 10 mL of DMEM/F12 medium. Afterward, the supernatant was replaced with 5 mL of plating neurobasal medium (neurobasal medium, 2% B-27 ™ supplement (50X), 1% 200 mM L-glutamine, 1% 100x P/S, 0.07% 10 mM β-mercaptoethanol, and 0.125 mM L-glutamic acid). The cortices were mechanically broken down in the medium by pipetting 3–5 times with a 10 mL pipette and a glass Pasteur pipette. All tissues were broken down, and the cells were distributed homogeneously in the medium; dead cells and tissue pieces were left for 5 min to settle. Then the supernatant was separated, and cells were seeded at 104 cells/well in PDL-coated 96-well plate culture dishes. Each well was subjected to the medium change in half on day three with AraC Medium (Neurobasal Medium, 2% B-27 ™ supplement (50X), 1% 200 mM L-glutamine, 1% 100x penicillin/streptomycin, 0.07% and 25 μM 10 mM Cytosine β-D-arabinofuranoside). On the 4th day, the medium places in each well were replaced with feeding neurobasal medium (neurobasal medium, 2% B-27 ™ supplement (50X), 1% 200 mM L-glutamine, 1% 100x penicillin/streptomycin, 0.07%) with a rate of 90%. Later, the medium was changed with feeding neurobasal medium every 3 or 4 days for 8 days until the neurons matured.

### 2.4. Primary cortical neuron characterization

Immunocytochemical staining was performed using the anti-MAP2 antibody targeting the neurons’ cytoskeleton to characterize the neurons obtained with primary culture. Neurobasal medium was removed, and mature neurons were washed with phosphate buffer saline (PBS) and fixed with −20 °C methanol for 5 min at room temperature. Subsequently, the permeability of the cells was achieved with 0.1% Triton X-100 for 3 min at room temperature. After blocking was performed with the blocking solution, the cells were incubated at 4 °C overnight with the primary antibody (mouse monoclonal anti-MAP2) at 1:2000 concentration. The cells were then incubated with the secondary antibody (goat anti-mouse IgG-TR) for 1 h at room temperature. Counterstaining was done by using 4′,6-diamidino-2-phenylindole (DAPI).

### 2.5. Concentration trials

Concentration trials were performed to determine the working concentration and possible toxic effects of A-T, A-T3, and Dimethyl sulfoxide (DMSO) and the lethal effects of Aβ on neurons. DMSO was used as a solvent for A-T, A-T3, and Aβ. We determined the concentrations as 50 μM, 100 μM, and 200 μM for A-T and 5 μM, 10 μM, and 20 μM for A-T3, then applied the solutions to the cells for 24 h (Osakada et al., 2004; Then et al., 2009). DMSO was used at 0.1%, and Aβ at 2 μM concentration (Yang et al., 2010; Stine et al., 2011).

### 2.6. Establishment of two-dimensional in vitro neuroprotection model

**Figure 1:**
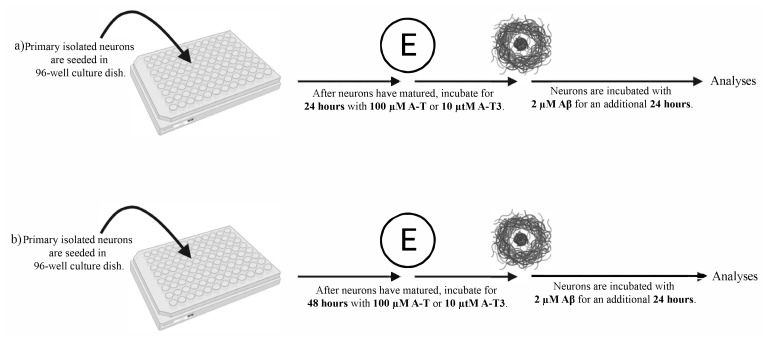
Overview of the experimental setup. Primarily isolated neurons were seeded in 96-well plates. After they matured, A-T and A-T3 were applied for 24 (a) or 48 (b) h. Then, cells were incubated with Aβ for additional 24 h without removing A-T and A-T3. The established experimental groups were analyzed at the end of the incubation process.

To investigate and compare the neuroprotective effects of A-T and A-T3 derivatives of vitamin E against the Aβ-induced neurodegeneration, we formed an in vitro neurodegeneration model. In this context, neurons were treated with previously tried and physiologically applicable concentrations of A-T and A-T3 for 24 or 48 h before Aβ was applied (Osakada et al., 2004; Then et al., 2009.). Then, 2 μM of Aβ was applied for 24 h. In brief, before Aβ administration, neurons were treated with 100 μM A-T and 10 μM A-T3 for 48 and 72 h (Figure 1).

After this process, cell viability, cytotoxicity, apoptosis, necrosis, amount of Aβ accumulation, and expressions of BACE1 and Cacna1c were measured with established methods.

### 2.7. Examination of cell viability and cytotoxicity

The MTT assay is a standardized colorimetric cell viability assay that determines the ability of the living cells to form MTT formazan crystals with succinate dehydrogenase enzyme activity (Durukan et al., 2019). For performing the MTT assay, the culture medium on neurons was removed, and cells were rinsed with PBS. Serum-free medium containing 10% MTT solution was added on top of the cells and incubated for 4 h at 37 °C, with 5% CO_2_. Then, the MTT solution was removed, and isopropanol was added to solve the formazan crystals and obtain a violet-like color. The absorbance values were measured at 570 nm wavelength via the ELISA microplate reader (μQuantTM, BiotekW Instruments Inc, USA). We have obtained the ratio of cell viability by calculating the percentages of the experimental groups compared to the positive control (Equation 1).


(Equation 1) $$Cellular\;Viability\;(\%)=\frac{Treated\;Group\;Absorbance}{Control\;Group\;Asorbance}\times 100$$

The neutral red assay is an assay that uses the ability to incorporate and bind the neutral red dye into lysosomes of living cells. This cytotoxicity test, used in many biomedical applications, enables quantitative analysis of the number of live cells and cytotoxicity in cell culture (Repetto et al., 2008). For the neutral red assay, the culture medium on the cells was removed, and the cells were rinsed with PBS. Then, the serum-free medium containing 10% neutral red was applied to the cells at 37 °C for 3 h. After incubation, the neutral red medium was removed, and the cells were washed with PBS. The fixation was performed with a solution containing 0.1% CaCl2 and 0.5% formaldehyde. After fixation, we added a solution containing 1% acetic acid and 50% ethanol, and the cells and cells were incubated at 37 °C for 10 min to extract the dye. After the incubation, the absorbance values of the samples were measured on the ELISA microplate reader (μQuantTM, BiotekW Instruments Inc, USA) at 490 nm. The cytotoxicity ratio was obtained by calculating the percentages of the experimental groups compared to the positive control (Equation 2).


(Equation 2) $$Cytotoxicity\;(\%)=\frac{Control\;Group\;Absorbance-Treated\;Group\;Absorbance}{Control\;Group\;Absorbance}\times 100$$

### 2.8. Demonstration of Aβ accumulation with Congo red dye

We used Congo red dye to show the Aβ accumulation in the groups treated with 2 μM Aβ. Media from cells treated with Aβ was removed. After washing with PBS, cells were fixed with cold 100% methanol for 5 min at room temperature. After fixation, nucleus staining was done with hematoxylin. Then, 0.1% Congo red solution was applied to the cells. After staining was finished, Congo red residues were removed by washing with PBS, and stained Aβ fragments were observed with invert-fluorescence microscopy (Olympus IX70 Inverted Microscope, Japan). Then, the percentage of areas stained with Congo red dye was measured using Image J (National Institutes of Health, USA).

### 2.9. Acridine orange/propidium iodide staining

AO and PI are fluorochromes bound to DNA and emit green and orange fluorescence. While AO crosses the membrane of living and early apoptotic cells and causes the green nuclei and intact structure or bright green nucleus, respectively, PI causes an orange nucleus on late apoptotic and necrotic cells (Abdel Wahab et al., 2009). Neurobasal medium was removed from cells, and the cells were rinsed with PBS. Then they were stained with the 1:1 mixture of AO:PI for 1 min. Dye was removed from the cells, and cells were rinsed twice with PBS. Then, imaging was performed with inverted microscopy (Olympus IX70 Fluorescence Microscope, Japan), and apoptotic and necrotic cells were counted via Image J (National Institutes of Health, USA).

We have performed counting following the previous studies. We counted:

-Uniform green cells with organized structure as living cells,
-Cells with bright green areas of chromatin condensation as early apoptotic cells,
-Cells that have dense orange chromatin condensation as late apoptotic cells,
-Cells that are orange and have intact nuclei as necrotic cells.

We examined three different images to count the cells for each group, and apoptotic and necrotic cells were calculated via Equations 3 and 4 (Ciapetti et al., 2002; Abdel Wahab et al., 2009).


(Equation 3) $$Apoptotic\;Cells\;(\%)=\frac{Total\;number\;of\;apoptotic\;cells\;(early\;or\;late)}{Total\;count\;cell}\times 100$$

(Equation 4) $$Necrotic\;Cells\;(\%)=\frac{Total\;number\;of\;necrotic\;cells}{Total\;count\;cell}\times 100$$

### 2.10. Real-time polymerase chain reaction analysis

To examine the protective effects of A-T and A-T3 against Aβ-induced neurodegeneration at the molecular level, CACNA1c, which controls calcium channels, and BACE1, which is related to the metabolism of APP protein, were selected (Bhat et al., 2012; Das and Yan, 2017).

Primers of about 20 bases were explicitly designed for CACNA1c and BACE1 genes using the Primer3 version V4.0 (Howard Hughes Medical Institute, USA) program and purified by the desalting method. To prevent DNA contamination, disposable columns and pipette tips were used, and sterile laboratory environment conditions were provided. The primers were designed according to the cDNA sequences. Cells were analyzed for the CACNA1c and BACE1 gene expressions using the designed primers. The sequences of the primers used in the study are given in Table 1. The design and synthesis steps of the primers were carried out by BMLabosis BM Lab. Sist. Ltd. Şti. (Ankara, Turkey).

In order to perform real-time polymerase chain reaction analysis (RT-PCR), RNA was isolated from the cells, the isolated RNAs were converted into complementary DNA (cDNA), and the expression levels of the relevant gene regions were examined by RT-PCR analysis.

RNA was isolated from cells using the TRI reagent protocol. In this context, neurons were collected by using TRI Reagent. Then 0.2 mL of chloroform was added for every 1 mL of TRI reagent used for forming a three-phase formation in the tubes. Since the RNA content is present in the uppermost colorless aqueous phase, this phase was separated, and 0.5 mL of isopropanol was added for every 1 mL of TRI reagent used to isolate the RNA purely. After incubation at room temperature, the samples were centrifuged at 2–8 °C for 10 min at 12,000 RCF. Ethanol (75%) was on the samples, and the samples were centrifuged. The supernatant was removed for the RNA to dry. After drying, the samples were diluted with 250 μL nuclease-free water.

The total RNA concentration was calculated with a NanoDrop device (NanoDrop Q 5000 UV-Vis spectrophotometer, Quawell Technologies Inc, China). Conforming to the manufacturer’s protocol, a sufficient amount of RNA was used to synthesize cDNA with a Transcriptor High Fidelity cDNA Synthesis Kit (Roche Molecular Systems, Inc, USA), and RT-qPCR was performed with a LightCycler^®^ 480 SYBR^®^ Green I Master (Roche Molecular Systems, Inc, USA). The glyceraldehyde 3-phosphate dehydrogenase (GAPDH) gene was used as an internal control for relative expression analysis.

### 2.11. Statistical analysis

All results were evaluated statistically with GraphPad Prism version 5.0 (GraphPad Software Inc., San Diego, CA, USA) and represented as the mean ± standard deviation (SD). We compared the statistical significance levels between different groups such as control, Aβ, A-T + Aβ, and A-T3 + Aβ with the one-way analysis of variance (ANOVA) post-test of Tukey’s multiple comparison test, and p-values less than 0.05 were considered statistically significant.

## 3. Results

### 3.1. Primary neuron isolation, culturing, and characterization

**Figure 2:**
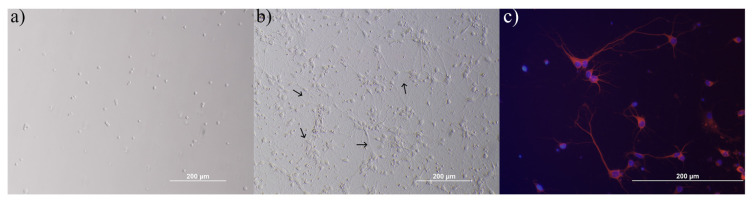
0-day (a), 8-day (b), and immunolabeled (c) images of neurons. Neurites are shown with the arrow (↑) sign. Immunolabeled images were captured at 20× magnification, while other images were captured at 10× magnification. Immunolabeled images of neurons obtained from the primary isolated neurons were labeled with anti-MAP2 antibody. Primary antibody: mouse monoclonal Anti-MAP2 (1: 2000 concentration), secondary antibody: goat antimouse IgG-TR (1: 100 concentration), cell nuclei: blue (Marked with DAPI), somas and axons: red.

Neurons isolated from 17–18-day-old rat embryos were incubated in feeding neurobasal medium. After 8 days, increased cell size and development of long and distinct neurites were observed. In these neurons, rounded cell bodies were decreased and changed to the neuronlike morphology. At this point, they were considered mature (Figures 2a and 2b). No passaging process has been carried out on the neurons.

After the neurons matured, they were characterized using anti-MAP2 antibody via fluorescence staining method before the experimental stage. We showed that this neuron culture was almost pure neuronal culture (Figure 2c).

### 3.2. Measuring cell viability and cytotoxicity

**Figure 3:**
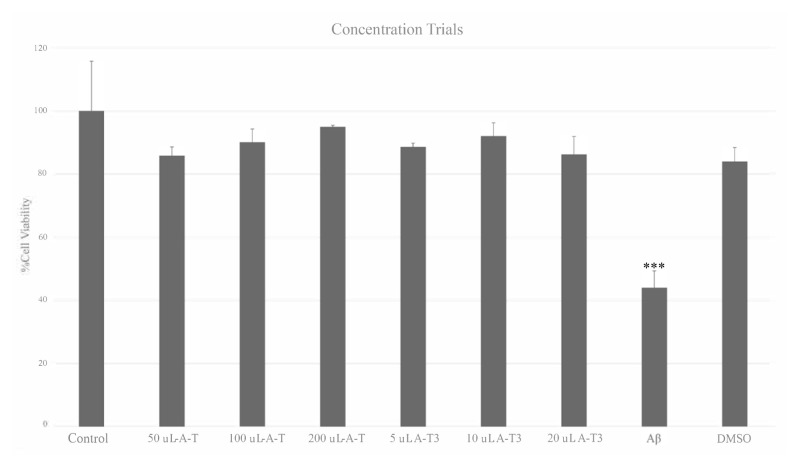
The concentration trials of Aβ, DMSO, and three different doses of A-T and A-T3; all groups were compared statistically against the control group; Vitamin E derivatives and DMSO did not cause any adverse effects on cell viability. Cell viability was measured significantly lower in the Aβ application group (***: p ≤ 0.001)

In concentration trials, it was revealed that the determined doses of DMSO, A-T, and A-T3 did not have a significant toxic effect on neurons, and the 2 μM Aβ significantly decreased the cell viability on 24 h of application. After 24 h of Aβ administration, cell viability was significantly decreased to 56% (p ≤ 0.001), and cytotoxicity was observed on 65% (Figure 3).

**Figure 4:**
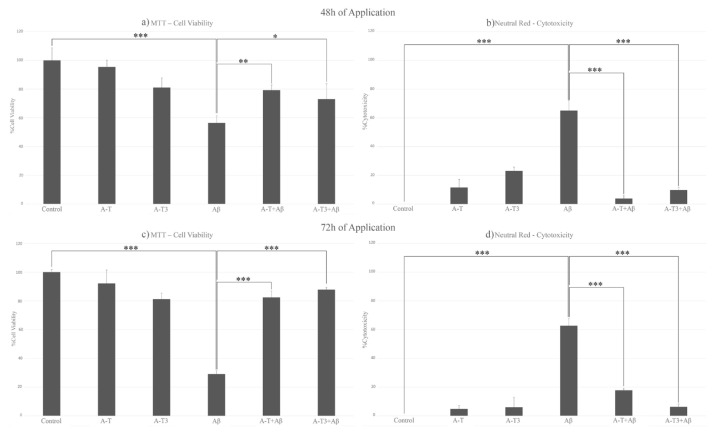
The cytotoxicity and viability values of neurons after the neuroprotection model have been established (a, b, c, d). Cell viability decreased significantly with the Aβ application. Cell viability was significantly higher in the groups in which vitamin E derivatives and Aβ were applied together than in the group in which Aβ was applied alone. a) Cell viability % values of 48-h application groups, b) cytotoxicity % values of 48-h application groups, c) cell viability % values of 72-h application groups, d) cytotoxicity % values of 72-h application groups (*:p ≤ 0.05, **:p ≤ 0.01, ***: p ≤ 0.001), control: group with nothing applied, A-T: 100 μM of A-T-treated group, A-T3: 10 μM of A-T3-treated group, Aβ: 2 μM of Aβ-treated group, A-T+Aβ: 48 or 72 h A-T- and 24 h Aβ-treated group, A-T3+Aβ: 48 or 72 h A-T3- and 24 h Aβ-treated group.

A statistically significant decrease was not observed in the A-T-treated group for 48 h in cell viability, and it was observed at 95% at the end of the 48 h of application. However, at this time section in the A-T3-treated group, cell viability was decreased by 81%. In the groups treated with both Aβ and one of the vitamin E derivatives, the cell viability was significantly increased, and the rate of cytotoxicity was observed to be significantly lower compared to the group treated with only Aβ. Cell viability exceeded 70% in these groups, and cytotoxicity was observed below 10%. Comparing the A-T and A-T3’s protective effects, cell viability was significantly increased to 79% in the A-T + Aβ group (p = 0.00290) and 73% in the A-T3 + Aβ group (p = 0.03043) when compared with the group that treated with only Aβ. Cytotoxicity values were observed as 4% in the A-T + Aβ group and 10% in the A-T3 + Aβ group, and the p-values were measured below 0.001. No statistically significant difference was observed between these two groups in terms of percent cell viability and percent cytotoxicity values (Figures 4a and 4b).

Cell viability did not significantly decrease in 72 h of application of A-T, and it was observed at 92%. Nevertheless, cell viability was slightly reduced in the A-T3 treatment group, and it was at 81% again. Derivatives of vitamin E never reduced cell viability below 70% in any experimental groups. In the groups treated with both Aβ and vitamin E derivatives, the cell viability was significantly higher than those treated with only Aβ, and p-values in these groups were always measured to be below 0.001. The rate of cytotoxicity also decreased significantly compared to the Aβ applied group. While cell viability was 82% in the A-T + Aβ group, it was 88% in the A-T3 + Aβ group. It was 17% in the A-T + Aβ group in cytotoxicity and 6% in the A-T3 + Aβ group. There was no statistically significant difference between these two groups regarding cell viability and cytotoxicity values (Figures 4c and 4d).

### 3.3. Comparing the amounts of Aβ accumulation

**Figure 5:**
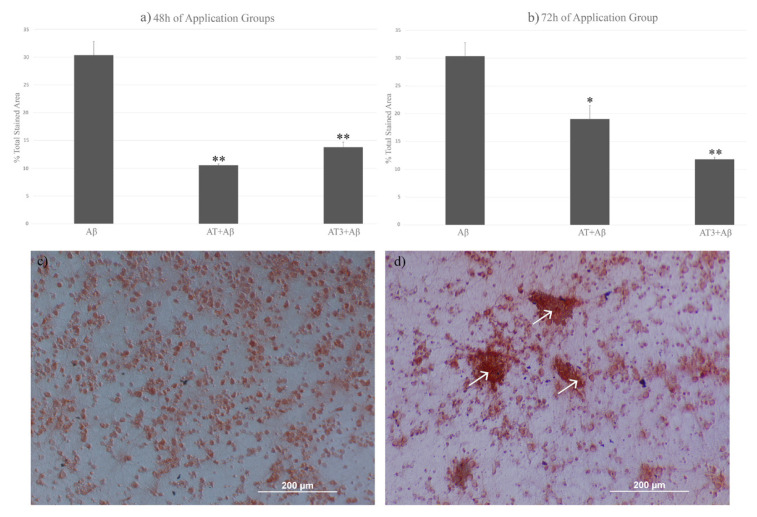
Congo red staining of Aβ accumulation in the neuron culture and comparison of Aβ accumulation in Aβ, A-T + Aβ, and A-T3 + Aβ groups. More than 25% of the total area is marked with Congo red in photographs taken in the Aβ application group. In the A-T+Aβ and A-T3+Aβ groups, the marked area was observed below 15%. Arrow (↑) sign was used to show Aβ accumulations. a) Comparison of Aβ accumulation amounts in 48-h groups, b) comparison of Aβ accumulation amounts in 72-h groups, c) Congo red staining of the group without Aβ application, d) Congo red staining of the group with Aβ application. (All groups compared statistically against the Aβ group, *: p ≤ 0.05, **: p ≤ 0.01, ***: p ≤ 0.001).

Congo red staining was performed to observe the Aβ accumulation. The regions stained with Congo red were analyzed using Image J (National Institutes of Health, USA). In the group treated with only 2 μM Aβ, Aβ plaques accumulated in 30% of the area marked with Congo red. This accumulation showed a significant decrease with 48 and 72 h of applications of A-T and A-T3. The amount of Aβ accumulation was analyzed as 10% in 48 h of application of A-T (p = 0.00152) and 14% in A-T3 (p = 0.00304). In the 72-h application, the amount of Aβ accumulation was observed as 19% in the A-T group (p = 0.03391) and 12% in the A-T3 group (p = 0.00578). No statistically significant difference was found between A-T and A-T3 groups regarding percentage Aβ accumulation in both 48 and 72 h of application (Figure 5).

### 3.4. Comparing the real-time polymerase chain reaction analysis

**Figure 6:**
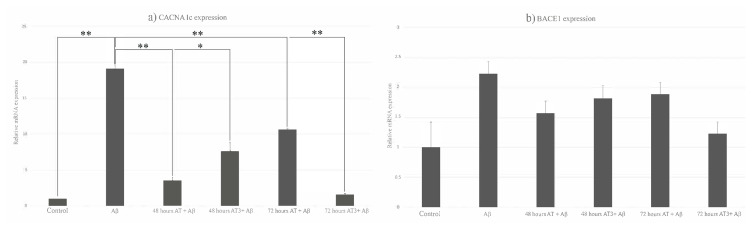
Relative CACNA1c and BACE1 mRNA expression levels relative to GAPDH control expression. CACNA1c expression and BACE1 expression increased with Aβ administration. In the groups in which vitamin E derivatives and Aβ were applied together, the expression amounts of CACNA1c and BACE1 genes were lower than those in the group in which only Aβ was applied. a) Relative expression of CACNA1c, b) relative expression of BACE1, (*: p ≤ 0.05, **: p ≤ 0.01), Aβ: 2 μM of Aβ-treated group, A-T+Aβ: 48 or 72-h A-T- and 24-h Aβ-treated group, A-T3+Aβ: 48 for 72 h A-T3- and 24-h Aβ-treated group.

RT-PCR results were analyzed by the delta Ct method and normalized with the GAPDH housekeeping gene. After normalization, the expression amounts of CACNA1c and BACE1 genes were calculated. When the amount of gene expression was examined, it was observed that CACNA1c expression in neurons significantly increased when treated with 2 μM Aβ for 24 h, and p-value was measured at 0.00565 value. A significant decrease was observed in Aβ + A-T (p = 0.00739) and A-T3 + Aβ (p = 0.02095) groups in terms of CACNA1c expression at 48 h of application. The relative CACNA1c expression also significantly decreased in the A-T + Aβ group at 72 h of application (p = 0.014665). Besides the control group, the lowest expression of CACNA1c was observed in the A-T3 + Aβ group (p = 0.00593) at 72 h of application (Figure 6b). Even though different amounts of expressions have occurred between Aβ + A-T and A-T3 + Aβ groups in different periods, there was no statistically significant difference between these groups. Even if an increase in the amount of the BACE1 gene was observed after 2 μM Aβ administration for 24 h (p = 0.060754), this increase was not statistically significant. With the A-T and A-T3 applications, the increased expression levels of BACE1 were decreased (Figure 6b).

### 3.5. Apoptotic and necrotic cell staining

Apoptosis and necrosis percentages were examined using Image J (National Institutes of Health, USA). It was found that necrosis was not observed in neurons in the control group. In addition, only a meager percentage of early apoptosis (11%) and late apoptosis (2%) were observed in these groups. There was an increase in late apoptosis and necrosis percentages in the group where Aβ was applied. As a result of the analysis, 63% of the total cell percentage was observed as necrotic, 11% as early apoptotic, and 19% as late apoptotic cells after 2 μM of Aβ application for 24 h. For the groups treated for 48 h, when A-T and Aβ were applied together, the percentage of cells showing apoptosis and necrosis was calculated as 28% and 4%, respectively. In the A-T3 + Aβ group, the amount of apoptosis is 17%, while the amount of necrosis is 22%. In the groups treated for 72 h, 16% apoptotic cells and 19% necrotic cells were observed in the A-T + Aβ group, while the percentage of apoptotic cells did not change in the A-T3 + Aβ group, and the percentage of necrotic cells was calculated as 10% (Table 2).

## 4. Discussion

Neurodegeneration can be defined as the loss of structure and function of neurons. Aβ, a protein fragment, is the most common cause of Alzheimer’s disease and neurodegeneration. Although Aβ causes a toxic effect on neurons, studies show that antioxidants such as vitamin E could lower these harmful effects (Sen et al., 2006; Chang et al., 2018; Gugliandolo et al., 2019). The antioxidant effects of vitamin E come from its radical scavenging feature. In addition to its antioxidant properties, vitamin E is an essential nutrient for the brain. Studies have shown that due to vitamin E deficiency, antioxidant protection was not sufficient to prevent neurodegeneration. Vitamin E, as well as other biochemical factors, exerts therapeutic or protective effects against Aβ-induced neurodegeneration by reducing neurons’ oxidative stress (Ulatowski and Manor, 2015; Gugliandolo et al., 2017). Yatin et al. (2000) showed that pretreatment of 50 μM A-T prevents Aβ accumulation and ROS formation on hippocampal neurons. It was also revealed that decreased plasma vitamin E levels are related to the future threat of developing neurodegenerative diseases such as Alzheimer’s (Yatin et al., 2000). Parallel to previous studies, we also showed that A-T and A-T3 were protective against Aβ-induced neurodegeneration. Additionally, the protective effects of A-T3 against neurodegeneration might be higher than A-T in more extended application periods.

Vitamin E prevents Aβ from accumulating in the intercellular region to form plaques and the emergence of Aβ-induced abnormalities (Mazur-Kolecka et al., 2006). Ibrahim et al. (2021) showed that both A-T and A-T3 affected the Aβ aggregation. While A-T reduces the Aβ aggregation in high concentrations, A-T3 can reduce it in much lower concentrations (Ibrahim et al., 2021). However, in another study, it has been shown that the usage of vitamin E at higher concentrations may cause a decrease in cell viability and increase cytotoxicity. When A-T was applied at 200 μM and above concentration, cell viability in neurons decreased by 40%, and cytotoxicity increased above 20% (Then et al., 2009). In the light of these studies and our concentration trials, we decided to use A-T and A-T3 at 100 μM and 10 μM concentrations, respectively. A significant decrease in the amount of Aβ-induced cytotoxicity and necrosis was observed with the application of A-T and A-T3. Some studies show that A-T or A-T3 applications can reduce the amount of Aβ accumulation (Yang et al., 2010; Gugliandolo et al., 2019; Ibrahim et al., 2021). Parallel with these studies, we also measured the amount of Aβ accumulation as significantly lower in the A-T and A-T3 application groups. This informs us that the A-T and A-T3 isoforms of vitamin E show their protective effects on neurons by reducing the amount of Aβ accumulation and reducing cytotoxicity. We also showed that A-T and A-T3 protect cells from the unprogrammed form of cell death, necrosis, by preventing Aβ-induced stress. When these results and previous studies in the literature are evaluated together, it can be concluded that A-T and A-T3 act as protective agents against Aβ-induced stress. Our results also suggest that A-T3 can act at lower concentrations against Aβ accumulation, thus making it a better protective agent than A-T. Besides, A-T and A-T3 also showed a regulatory effect on CACNA1c and BACE1 genes.

We found that treatment with A-T and A-T3 prior to Aβ administration has a statistically significant reducing effect on increased CACNA1c expression on neurons. As described by Sciacca et al. (2018) and Berridge (2010), defects in the intracellular mechanisms that regulate calcium channel influx that normally regulates neuron functioning cause deterioration in neurons. Studies show that Aβ accumulation can disrupt the calcium signaling pathway and affect the calcium balance in the cell. Previous studies show that vitamin E may influence the mechanisms that regulate the calcium channel influx of neurons besides its antioxidant effects (Berridge, 2010; Bhat et al., 2012 Sciacca et al., 2018). Similarly, the A-T and A-T3 applications reduced the CACNA1c expressions of the neurons by 50%–75% compared with the Aβ treatment group. This suggests that the A-T and A-T3 can prevent the expression of calcium channels and keep neurons’ calcium levels at homeostasis. Vitamin E also reduces the BACE1 expression by reducing the amount of cholesterol (Grimm et al., 2016; Das and Yan, 2017). Parallel with these studies; we also measured a decrease in the expression of the BACE1 gene. However, our results for BACE1 expression were not statistically significant.

A-T, the most basic form of vitamin E, protects neurons against Aβ-induced neurodegeneration (Yatin et al., 2000; Sung et al., 2004; Lloret et al., 2019). However, according to other studies, A-T3 could be more effective against neurodegeneration than A-T. It shows more neuroprotective effects in nanomolar concentrations than A-T. In addition, the A-T3-rich fraction isolated from palm oil reduces the Aβ oligomerization and can be used as an alternative treatment against Aβ-induced neurodegeneration (Khanna et al., 2006; Ibrahim et al., 2017). It is also shown that 10 μM of A-T3 protected neurons better against H_2_O_2_-induced cell death than the same amount of A-T (Osakada et al., 2004). Based on these studies, we compared A-T and A-T3 against Aβ-induced neurodegeneration and observed different amounts of protective effects on neurons treated with A-T and A-T3 at different periods. In 48 h of application, cell viability in A-T + Aβ group was higher, and cytotoxicity was lower when compared with A-T3 + Aβ group. In addition, the amount of Aβ accumulation was observed to be lower in this group. We also observed a difference in the percentages of necrotic cells between the A-T + Aβ and A-T3 + Aβ groups. As shown in Table 2, A-T3 was more successful than A-T in protecting cells against Aβ-induced necrosis within 72 h of application. In parallel with cytotoxicity, amount of Aβ accumulation, and RT-PCR results, the effect of A-T3 is less in 48 h of application compared to 72 h of application. In the A-T group, this situation is the opposite. As claimed by a study, oxidative stress induces necrosis by causing mitochondrial hyperpolarization (Choi et al., 2009). Thus, in more extended application periods, A-T3 might be decreasing the necrosis more than A-T due to its oxidative stress inhibiting effects. It could be interpreted that when comparing low concentrations of A-T3 with high concentrations of A-T, it not only prevents Aβ-induced necrosis but can also increase its protective effect in more extended periods of application.

Grimm et al. (2016) claim that A-T and A-T3 have similar effects on neurodegeneration in preventing ROS formation. However, tocopherols and tocotrienol might be inducing the amyloidogenic pathway of APP and could increase Aβ production (Grimm et al., 2016). Despite that, Ibrahim et al. (2021) stated in their study that both A-T and A-T3 prevent the aggregation of Aβ. While A-T is effective at high concentrations, A-T3 is effective at lower and higher concentrations (Ibrahim et al., 2021). Concerning our results, in both 48 and 72 h of application effectiveness, A-T and A-T3 were close to each other. They both prevented Aβ accumulation in the extracellular region and increased the viability of neurons via regulating apoptosis, necrosis, and gene expressions. However, at 72 h of administration, an increase in the efficacy of A-T3 was consistently observed in each analysis, while the efficacy of A-T remained constant. For example, we observed that the expression of CACNA1c and BACE1 genes decreased more in the A-T3 group compared to A-T at this period. However, the differences between the neuroprotective effects of A-T and A-T3 were not statistically significant.

In conclusion, there is a difference between the protective effects of A-T and A-T3 against Aβ-induced neurodegeneration. Even though the measured differences were not statistically significant, these results indicate that the protective effects of A-T3 may increase in long-term applications compared to A-T. In order to investigate this situation, future studies including longer application times of A-T3 and examining the effects of these forms of vitamin E against the mechanism of Aβ pathology may be conducted.

## Figures and Tables

**Table 1:** Primer sequences of target genes.

Target gene	Primer type	Primer sequence (5′ – 3′)	Scale of synthesis (nmol)	Number of bases	TM (°C)	GC (%)	MW (g/mol)	nmol	NCBI Accession “
CACNA1c	Forward	CTCCAGTTGCCTGTCTGAGG	50	20	61	60	6100	59	204
	Reverse	GCTCCCATAGTTGGAACCTCC	50	21	62	57	6342	76	
BACE1	Forward	ATGGCTTTTGGCTAGGGGAG	50	20	59	55	6244	66	201
	Reverse	TGGCCGTAGGTATTGCTGAG	50	20	59	55	6204	77	
GAPDH	Forward	TTGTGCAGTGCCAGCCTC	50	18	58	61	5467	64	202
	Reverse	ATGAAGGGGTCGTTGATGGC	50	20	59	55	6253	59	

**Table 2:** Apoptosis % and necrosis % rates calculated by AO/PI staining at 48 and 72 h of applications.

Group	Apoptosis and necrosis (%)	48 h	72 h
Control	Early apoptosis	11.32	11.40
	Late apoptosis	2.83	2.85
	Necrosis	0	0
Aβ	Early apoptosis	11.54	11.37
	Late apoptosis	19.23	19.48
	Necrosis	63.46	63.46
A-T + Aβ	Early apoptosis	19.44	13.76
	Late apoptosis	9.26	3.67
	Necrosis	3.70	19.27
A-T3 + Aβ	Early apoptosis	9.29	9.17
	Late apoptosis	7.14	8.33
	Necrosis	22.86	10.83
